# Risk-reducing mastectomy rates in the US: a closer examination of the Angelina Jolie effect

**DOI:** 10.1007/s10549-018-4824-9

**Published:** 2018-05-28

**Authors:** Alexander Liede, Mona Cai, Tamara Fidler Crouter, Daniela Niepel, Fiona Callaghan, D. Gareth Evans

**Affiliations:** 1 Center for Observational Research, Amgen Inc., Thousand Oaks, CA USA; 2 SimulStat Incorporated, Solana Beach, CA USA; 3 Global Medical Affairs, Amgen GmbH, Zug, Switzerland; 4 Genomic Medicine, Manchester Academic Health Science Centre, The University of Manchester, Manchester, UK; 5 Central Manchester University Hospitals NHS Foundation Trust, Saint Mary’s Hospital, Oxford Road, Manchester, M13 9WL UK

**Keywords:** Bilateral mastectomy, Genetic testing, BRCA mutation

## Abstract

**Purpose:**

In 2013, Angelina Jolie disclosed in the *New York Times (NYT)* that she had undergone risk-reducing bilateral mastectomy (RRBM) after learning that she was a *BRCA1* mutation carrier. We examined the rates of *BRCA* testing and RRBM from 1997 to 2016, and quantified trends before and after the Jolie op-ed.

**Methods:**

This observational study of insurance claims data representative of the commercially-insured US population (Truven MarketScan® database) measured *BRCA* testing and RRBM rates among females ≥ 18 years. Censoring events were breast cancer or ovarian cancer diagnosis, last follow-up date (September 2016), or death. Interrupted time series analyses were used to quantify trends before and after the op-ed.

**Results:**

Angelina Jolie’s *NYT* op-ed led to a statistically significant increase in the uptake of genetic testing and in RRBM among women without previous diagnosis of breast or ovarian cancer in the US population, and in women who did not undergo testing for *BRCA* (*P* < 0.0001 for both). The rate (slope) of RRBM among women who were previously tested for *BRCA* (*P* = 0.70) was unchanged. After excluding women with in-situ tumors, the editorial’s effect became less pronounced, suggesting that high-risk women with in-situ breast cancers were most influenced by Jolie’s announcement.

**Conclusion:**

The *Angelina Effect*—a term coined by *Time* magazine to describe the rise in internet searches related to breast cancer genetics and counseling—represents a long-lasting impact of celebrity on public health awareness as significant increases in genetic testing and mastectomy rates were observed and sustained in subsequent years.

**Electronic supplementary material:**

The online version of this article (10.1007/s10549-018-4824-9) contains supplementary material, which is available to authorized users.

## Introduction

On May 14, 2013, actress, filmmaker, and humanitarian Angelina Jolie disclosed in an open editorial (op-ed) to the *New York Times* entitled *“My Medical Choice”* that she had learned she was a *BRCA1* mutation carrier and had undergone a risk-reducing bilateral mastectomy procedure with reconstruction [1]. Women with inherited mutations in the *BRCA1* or *BRCA2* genes represent a population at greatest risk of developing early-onset breast cancer and ovarian cancer, and, therefore, face difficult decisions around whether to have risk-reducing surgery or opt for increased surveillance for early detection [2–6]. In her letter, Angelina Jolie reflected on her personal process in dealing with the implications, and emphasized that women should make their own informed choices.

“For any woman reading this, I hope it helps you to know you have options. I want to encourage every woman, especially if you have a family history of breast or ovarian cancer, to seek out the information and medical experts who can help you through this aspect of your life, and to make your own informed choices” [1]

Angelina Jolie’s decision generated publicity worldwide, and led to increased awareness and interest in hereditary breast cancer and genetic testing, including publications in the medical domain evaluating this Angelina Jolie Effect [7, 8]. In December 2016, Desai and Jena [9] published on mastectomy rates in the United States using a large representative health insurance database (MarketScan®, Truven Health Analytics), and reported that the Effect was not sustained over a period beyond 60 days. The Desai and Jena report had significant limitations including short follow-up time (monthly mastectomy rates for each of the 7 months following Ms. Jolie’s editorial). More importantly, the lack of censoring resulted in the inclusion of women with a diagnosis of cancer, including breast cancer and ovarian cancer, which may have meant that many therapeutic rather than prophylactic mastectomy procedures were included, diluting any trend. Using the identical MarketScan® data source, the objective of our study was to examine the rates of *BRCA* genetic testing and rates of risk-reducing mastectomies from 1997 to 2016, applying methods that censor upon diagnosis of breast and ovarian cancer, and to measure changes in these trends upon the Jolie op-ed publication on May 14, 2013.

## Methods

### Data source

For our analysis, we used the MarketScan^®^ Commercial Claims and Encounters and Medicare Supplemental and Coordination of Benefits databases (Truven Health Analytics, an IBM Company, Ann Arbor, MI). MarketScan databases represent a large and diverse sample of individuals in the United States who have employer-based health insurance plan from approximately 350 private payers. The de-identified patient-level data captures demographics, enrollment information, inpatient, outpatient, and prescription drug claims. Marketscan includes over 22.3 million individuals covered in 2016 calendar year, 8.6 million adult female covered lives, which represents approximately 7% of the overall adult female population in the U.S [10].

### Study population

We identified all females 18 years and older from January 1997 through end of follow-up (at the time of data analysis), September 2016. To ensure, we captured a population without cancer (cancers relevant to mastectomy as therapeutic intervention), censoring events were breast or ovarian cancer diagnosis, and administrative end of follow-up (September 2016), or death. All patients had a minimum of 12 months continuous enrollment prior to index date, defined as their baseline period. The index date was first enrollment date plus 365 days. If the individual had multiple continuous enrollment periods, the first enrollment period was used to capture earliest possible genetic testing or cancer diagnosis.

### Analysis

Descriptive analyses were performed for baseline characteristics: age, insurance type, geographic region, and median follow-up duration. Characteristics were provided by calendar periods—1997–2002, 2003–2012, 2013–2016, overall, and for never *BRCA* tested, and *BRCA* tested. *BRCA* cohorts were defined as those with *BRCA* testing during baseline or follow-up. If a patient was *BRCA* tested within 30 days of mastectomy, we considered this patient to be never *BRCA* tested—a conservative approach since we could not be certain from these administrative claims data whether the *BRCA* test was a factor in the decision to perform prophylactic mastectomy in this short time window.

We identified *BRCA1* and *BRCA2* genetic testing according to Current Procedural Terminology (CPT), Healthcare Common Procedural Coding System (HCPCS) codes 81211–81217 in outpatient claim. Mastectomy identified based on ICD-9/ICD-10 Procedure codes and CPT codes (listed in Online Resource 1). Breast cancer cases and ovarian cancer cases were censored and identified based on the following ICD-9 CM, or ICD-10 CM (International Classification of Diseases, Ninth or Tenth Revision, Clinical Modification) codes (Online Resource 2).

In separate sensitivity analyses, we censored for in situ breast cancers (ductal, lobular, or unspecified carcinoma in situ) (ICD-9 CM 233.0; ICD-10 CM D50.00-02, D05.90-92, D05.80-82, D05.10-12). We examined family history codes for breast and ovarian cancer (Z80.3, Z80.41, respectively) and considered performing further sensitivity analyses, but we determined that it was not feasible since < 1% of patients had any codes for a family history, and patients who did were already captured using previous *BRCA* testing.

Incidence rates (IR) of mastectomy were calculated monthly and trends over time were graphed—overall and by *BRCA* testing status (ever versus never tested), whereby the numerator included women who had a mastectomy during that month and qualified for inclusion in the denominator. Each qualifying patient only counted once and then they were removed from cohort. The denominator for a respective month represented the sum of person-time contributed by each female eligible that month up until mastectomy or censoring. Trends over time were evaluated by measuring incidence rate ratio (IRR) and 95% confidence intervals (CI). IRR was defined as month to month ratio of IR for mastectomies for each time period specified. IRs of *BRCA* testing were measured monthly and trends plotted over time, whereby the numerator represented who had evidence of at least one *BRCA* test during that month and qualified for inclusion in the denominator.

We used interrupted time series analyses [11] comparing slopes of incidence trends before and after the op-ed publication date in May 2013. The pre-intervention period was evaluated from January 1, 2003 through May 31, 2013 (right censor date for pre-intervention period used to keep monthly rate for May consistent), and the post-intervention period from June 1, 2013 through September 30, 2016. A quasipoisson regression model was used to model mastectomy incidence rates over time, allowing the model to fit different trend lines before and after May 2013. The quasipoisson regression model is a type of poisson model that is more robust to statistical assumptions and accounts for overdispersion. Autocorrelation was examined to assess the correlation between time points. Analyses were generated using SAS Software, version 9.4 (SAS Institute Inc., Cary, NC), and the time series analyses were performed using R software, version 3.3.2 (The R Foundation, Vienna, Austria).

## Results

The mean follow-up was approximately 2.8 years for overall cohort, median follow-up 2.0 years but was consistently longer among women who had undergone *BRCA* genetic testing versus non-*BRCA* tested for all calendar periods (Table 1). Women who had *BRCA* genetic testing were more likely to be younger, commercially insured, and residing in the Northeast compared with the non-*BRCA*-tested women. The mean age of women in the 1997–2016 study period was 43.5 (interquartile range, 25th and 75th percentiles [IQR]; 31;54). Mean age among those who had *BRCA* testing was 41.9 years (IQR 34;50); whereas the mean age was 43.5 years (IQR 31;54) for those who did not have testing for *BRCA* mutation in their patient data.

**Table 1:** Baseline characteristics of female beneficiaries by *BRCA* testing and time period (1997–2016) in the MarketScan database

	Characteristics	All				Ever BRCA tested				Non BRCA tested			
		1997–2016	1997–2002	2003–2012	2013–2016	1997–2016	1997–2002	2003–2012	2013–2016	1997–2016	1997–2002	2003–2012	2013–2016
Total N		46,364,847 (100.0)	3,045,858 (100.0)	34,127,783 (100.0)	9,191,206 (100.0)	121,480 (100.0)	3,709 (100.0)	79,618 (100.0)	38,153 (100.0)	46,243,367 (100.0)	3,042,149 (100.0)	34,048,165 (100.0)	9,153,053 (100.0)
Age	Mean (SD)	43.5 (15.9)	47.4 (17.3)	43.6 (15.8)	42.1 (15.5)	42.0 (10.8)	38.4 (8.5)	41.8 (10.4)	42.7 (11.6)	43.5 (15.9)	47.4 (17.3)	43.6 (15.8)	42.0 (15.5)
	Median (IQR)	43.0 (31.0–54.0)	46.0 (34.0–59.0)	43.0 (31.0–54.0)	41.0 (29.0–53.0)	42.0 (34.0–50.0)	39.0 (32.0–45.0)	42.0 (34.0–50.0)	42.0 (34.0–52.0)	43.0 (31.0–54.0)	46.0 (34.0–59.0)	43.0 (31.0–54.0)	41.0 (29.0–53.0)
Age group	18–34	15,220,699 (32.8)	775,173 (25.5)	11,011,382 (32.3)	3,434,144 (37.4)	32,714 (26.9)	1,225 (33.0)	21,108 (26.5)	10,381 (27.2)	15,187,985 (32.8)	773,948 (25.4)	10,990,274 (32.3)	3,423,763 (37.4)
	35–49	14,845,205 (32.0)	963,974 (31.6)	11,151,037 (32.7)	2,730,194 (29.7)	56,934 (46.9)	2,127 (57.3)	38,527 (48.4)	16,280 (42.7)	14,788,271 (32.0)	961,847 (31.6)	11,112,510 (32.6)	2,713,914 (29.7)
	50–64	12,272,751 (26.5)	795,550 (26.1)	9,073,954 (26.6)	2,403,247 (26.1)	30,590 (25.2)	352 (9.5)	19,496 (24.5)	10,742 (28.2)	12,242,161 (26.5)	795,198 (26.1)	9,054,458 (26.6)	2,392,505 (26.1)
	65–74	2,068,107 (4.5)	256,433 (8.4)	1,448,898 (4.2)	362,776 (3.9)	1,097 (0.9)	5 (0.1)	428 (0.5)	664 (1.7)	2,067,010 (4.5)	256,428 (8.4)	1,448,470 (4.3)	362,112 (4.0)
	75+	1,958,085 (4.2)	254,728 (8.4)	1,442,512 (4.2)	260,845 (2.8)	145 (0.1)	0 (0.0)	59 (0.1)	86 (0.2)	1,957,940 (4.2)	254,728 (8.4)	1,442,453 (4.2)	260,759 (2.8)
Insurance	Commercial	42,280,609 (91.2)	2,533,752 (83.2)	31,192,651 (91.4)	8,554,206 (93.1)	120,141 (98.9)	3,701 (99.8)	79,085 (99.3)	37,355 (97.9)	42,160,468 (91.2)	2,530,051 (83.2)	31,113,566 (91.4)	8,516,851 (93.0)
	Medicare	4,073,647 (8.8)	509,520 (16.7)	2,928,646 (8.6)	635,481 (6.9)	1,319 (1.1)	6 (0.2)	522 (0.7)	791 (2.1)	4,072,328 (8.8)	509,514 (16.7)	2,928,124 (8.6)	634,690 (6.9)
	Missing	10,591 (0.0)	2,586 (0.1)	6,486 (0.0)	1,519 (0.0)	20 (0.0)	2 (0.1)	11 (0.0)	7 (0.0)	10,571 (0.0)	2,584 (0.1)	6,475 (0.0)	1,512 (0.0)
Geographic regions	Midwest	10,439,354 (22.5)	934,730 (30.7)	7,846,230 (23.0)	1,658,394 (18.0)	21,136 (17.4)	852 (23.0)	14,991 (18.8)	5,293 (13.9)	10,418,218 (22.5)	933,878 (30.7)	7,831,239 (23.0)	1,653,101 (18.1)
	Northeast	7,764,864 (16.7)	397,152 (13.0)	5,468,414 (16.0)	1,899,298 (20.7)	30,288 (24.9)	454 (12.2)	19,066 (23.9)	10,768 (28.2)	7,734,576 (16.7)	396,698 (13.0)	5,449,348 (16.0)	1,888,530 (20.6)
	South	18,041,956 (38.9)	1,195,917 (39.3)	13,347,681 (39.1)	3,498,358 (38.1)	46,321 (38.1)	1,951 (52.6)	29,889 (37.5)	14,481 (38.0)	17,995,635 (38.9)	1,193,966 (39.2)	13,317,792 (39.1)	3,483,877 (38.1)
	West	9,071,156 (19.6)	442,880 (14.5)	6,670,023 (19.5)	1,958,253 (21.3)	21,673 (17.8)	446 (12.0)	14,035 (17.6)	7,192 (18.9)	9,049,483 (19.6)	442,434 (14.5)	6,655,988 (19.5)	1,951,061 (21.3)
	Missing	1,047,517 (2.3)	75,179 (2.5)	795,435 (2.3)	176,903 (1.9)	2,062 (1.7)	6 (0.2)	1,637 (2.1)	419 (1.1)	1,045,455 (2.3)	75,173 (2.5)	793,798 (2.3)	176,484 (1.9)
Follow Up months	Mean (sd)	33.6 (34.9)	73.3 (65.8)	34.5 (31.9)	16.7 (11.7)	52.3 (44.2)	186.0 (41.1)	60.9 (38.3)	21.3 (12.2)	33.5 (34.9)	73.2 (65.7)	34.5 (31.8)	16.7 (11.7)
	Median (IQR)	24.3 (12.1–43.6)	48.7 (18.2–121.6)	24.3 (12.1–48.7)	12.1 (8.1–24.3)	36.5 (21.2–73.0)	182.6 (161.3–220.1)	57.8 (31.4–82.1)	21.3 (11.1–33.4)	24.2 (12.1–43.6)	48.7 (18.2–120.7)	24.3 (12.1–48.7)	12.1 (8.1–24.3)

*SD* standard deviation, *IQR* interquartile range

The CONSORT [12] diagram of patient attrition based on the inclusion/exclusion criteria used to identify female beneficiaries with at least 1 year of continuous insurance coverage from January 1997 to September 2016 in the MarketScan database is provided in Online Resource 3. After meeting all inclusion/exclusion criteria, *N* = 533,753 women developed breast cancer and *N* = 81,427 women developed ovarian cancer during the follow-up period and were censored at that time.

### *BRCA* genetic testing

**Fig. 1:**
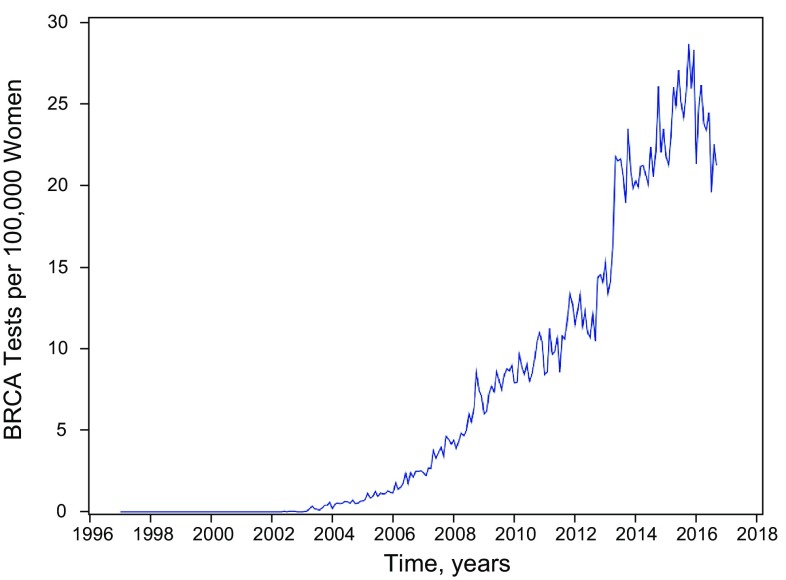
Monthly incidence rates of *BRCA* genetic testing among adult female enrollees in MarketScan database (1997–2006)

In these employer-based insurance claims data, the uptake of *BRCA* testing—among women without a previous diagnosis of breast or ovarian cancer—began in 2003 (Fig. 1). This may indicate a change in insurance coverage and reimbursement for genetic testing in 2003. An inflection in *BRCA* testing was evident after May 2013, as reported by Desai and Jenna [9]: the predicted IR jumped from 16.0 (95% CI 15.2–16.9) to 20.7 (95% CI 19.5–21.9) *BRCA* tests per 100,000 women from May to June of 2013 (*P* < 0.0001).

**Fig. 2:**
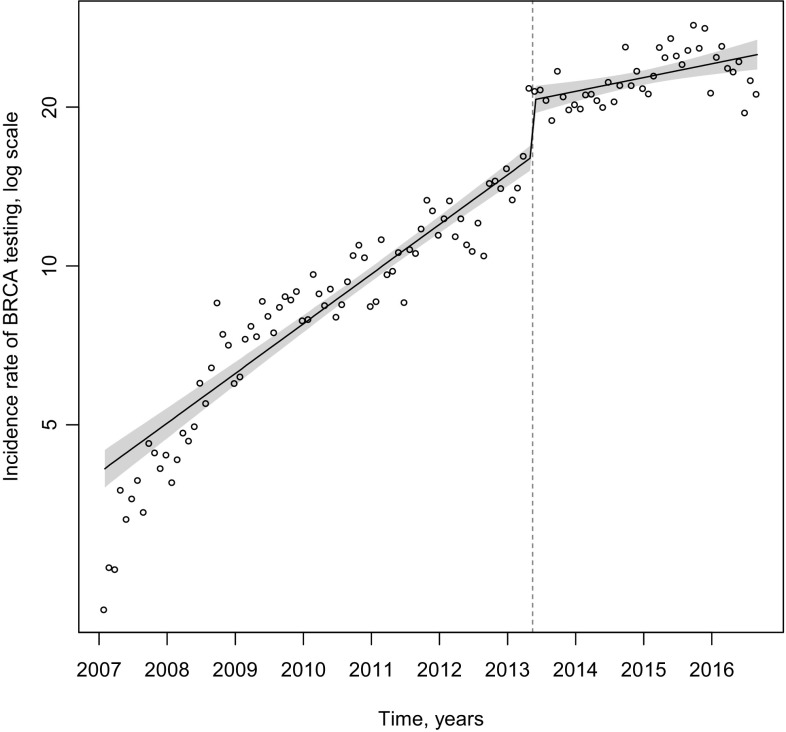
Interrupted time series analyses of *BRCA* testing monthly incidence rates per 100,000 among adult female enrollees in MarketScan database (2007–2016) before and after Jolie op-ed on May 14, 2013 (shading depicts 95% CIs)

Interrupted time series analysis examining the pre- and post-editorial IRs for *BRCA* testing indicated that there was an overall increase in *BRCA* testing after the editorial, but the IRR (slope) of *BRCA* testing decreased from a 2% increase in the IR each month to 0.5% increase (*P* < 0.0001) (Fig. 2).

### Risk-reducing mastectomy rates

**Fig. 3:**
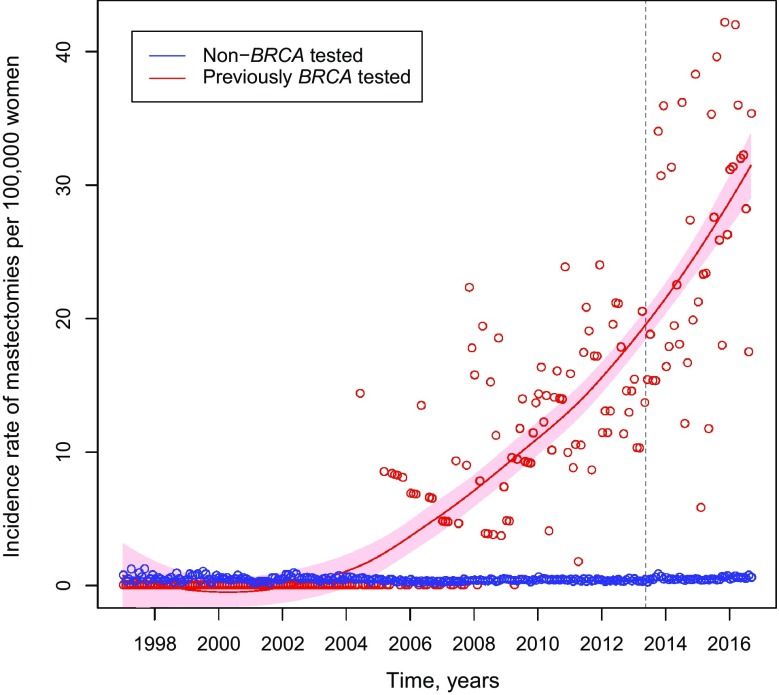
Monthly incidence rates of risk-reducing mastectomy procedures by *BRCA* testing among adult female enrollees in MarketScan database (2003–2016) (trend lines fitted using loess curves, and shading depicts 95% CIs)

In the 2003–2016 period, the association between the uptake of *BRCA* testing starting in 2003 and the steady increase in mastectomy procedures among women who were tested for *BRCA* mutations is illustrated (Fig. 3). Monthly mastectomy rates increased after 2007, with possibly a further increase in 2013. Mastectomy rates among women who did not undergo *BRCA* testing were comparatively low and constant during the study period; for example, 0.29 per 100,000 on September 01, 2006, and 0.56 per 100,000 on September 01, 2016.

**Fig. 4:**
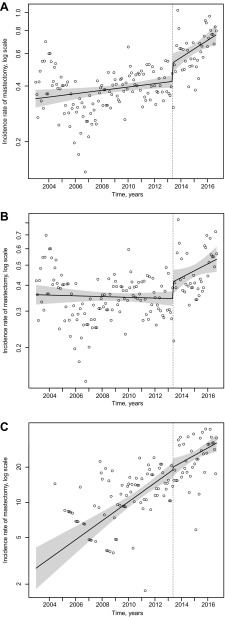
Interrupted time series analyses of risk-reducing mastectomy monthly incidence rates before and after Jolie op-ed on May 14, 2013 (shading depicts 95% CIs). **a** Incidence rates of risk-reducing mastectomy procedures per 100,000. **b** Incidence rates of risk-reducing mastectomy procedures among never *BRCA* tested per 100,000. **c** Incidence rates of risk-reducing mastectomy procedures among *BRCA* tested per 100,000

The interrupted time series with regression model revealed that, overall, there was a marked difference in the incidence rate of risk-reducing mastectomy (both slope and magnitude) before and after May 2013 (*P* < 0.0001, Fig. 4a). Specifically, there was an increase in the IRR after the Angelina Jolie editorial date, with IR of risk-reducing mastectomy increasing 0.2% each month before the editorial and 0.9% each month after May 2013 (*P* = 0.0112).

There was also a significant effect on the rates of risk-reducing mastectomy among women who did not undergo testing for *BRCA* (*P* < 0.0001) (Fig. 4b). Pre-intervention, the rate of mastectomies remained stable month over month; the IR was, if anything decreasing slightly by 0.04% each month (*P* = 0.61). Post-intervention, the rate of mastectomies increased each month by about 0.64% (*P* = 0.033).

Although the rate of mastectomy generally increased over time, the editorial did not have an impact on the rate of risk-reducing mastectomy for women who were previously tested for *BRCA* (*P* = 0.70) (Fig. 4c). Pre-intervention, the IR was increasing each month by 1.6%, compared to 1.2% each month post-intervention.

### Sensitivity analyses

We excluded patients with in-situ breast cancers in our censoring approach in separate analyses. Overall, the results were similar: the rates of prophylactic mastectomy increased significantly after the editorial for women who were not previously tested (*P* < 0.0001) and the combined (*BRCA* tested and non-tested) population (*P* < 0.0001). The estimates for the month-on-month IRs remained similar to the previous analyses, however, some of the *P* values changed: the differences in the slopes were no longer significant for women who were not previously *BRCA* tested (*P* = 0.28) and all women (*P* = 0.19). The results for women who were previously tested for the *BRCA* gene were similar to the previous analysis: the editorial did not have a significant impact on the rates of prophylactic mastectomy overall (*P* = 0.24) or on the slope of the IR (*P* = 0.09).

Finally, in order to correct for the violation of the autocorrelation in some of the models, we re-ran the interrupted time series models with robust variance estimates. Generally, we obtained similar results, except for the analysis of women who were not previously tested for *BRCA*. The estimates for the IRs were the same, but the change in slope of the IR before and after the editorial was no longer significant (*P* = 0.059).

## Discussion

This study describes that a large and immediate increase in *BRCA* testing was observed among commercially insured women 18 years of age and older in the United States following Angelina Jolie’s *New York Times* editorial published on May 14, 2013. Desai and Jenna reported similar findings using the same data source, but in contrast to these previous analyses, we examined a much longer time period spanning over 20 years (1997–2016) and, importantly, women were censored at breast or ovarian cancer diagnosis to accurately represent incidence of prophylactic or risk-reducing mastectomies among women and among those who have and have not had previous *BRCA* genetic testing. In the interrupted time series analyses, we report a statistically significant increase in risk-reducing mastectomy procedures pre- versus post-publication of the Jolie op-ed among all women and among those who had not undergone genetic testing.

In the MarketScan® employer-based insurance claims database, *BRCA* testing began in 2003, suggesting a seminal event relating to policy or access took place and led to an immediate uptake of genetic testing. In truth, Myriad Genetics, Inc. (Salt Lake City, UT) had launched a commercial full-length gene sequencing test for *BRCA1* and *BRCA2* in November 1996, outpacing the American Medical Association CPT codes used for health insurance reimbursement; CPT codes for genetic testing only went into effect in 2003 [13]. The introduction of new codes, which can result in misclassification bias, emphasizes the importance of recognizing the innate strengths and limitations of reimbursement claims databases such as MarketScan® or Medicare, [14] particularly when measuring trends in genetic testing or other interventions over time. Health insurance claims databases are also subject to changes in government policy affecting access and reimbursement. For example, the United States government introduced laws in recent years to prevent genetic discrimination from health insurers and employers: the Genetic Information Nondiscrimination Act (GINA) (2008), [15] the Affordable Care Act (2010), [16] and the GINA-required modification to HIPAA (2013). The introduction of these laws did not directly correspond with increases in *BRCA* testing rates in our study.

Risk-reducing mastectomy trends remained stable or unchanged for women who had a *BRCA* test; specifically, a steady increase was observed after May 14, 2013, but the slope in the rate of mastectomy procedures remained stable before versus after the Jolie op-ed. This finding suggests either that women who embarked on genetic testing, who have their cancer risk level well defined, were less influenced by the Jolie op-ed, or that the increased volume of individuals undergoing genetic testing included many women with a lower likelihood of having genetic predisposition and resultant lower proportion of positive test results. Based on the sensitivity analyses, the population of women most influenced by Jolie’s announcement may have been women with a diagnosis of in-situ carcinomas of the breast (ductal, lobular, or unspecified carcinoma in situ), considered stage 0 or a “pre cancer”. It is known that ambiguity around cancer risk—particularly among women with in-situ cancers, [17–20] women in high risk breast-ovarian cancer families who decline predictive testing [21, 22] or who receive uninformative results, [23, 24] or *BRCA* mutation carriers who do not proceed with risk-reducing surgeries [25–28]—can be associated with higher cancer-related distress and anxiety levels. A celebrity like Angelina Jolie announcing her decision to have a surgical procedure to prevent future cancer may have, to a larger extent, influenced these women facing a degree of uncertainty about future breast cancer risk to proceed more aggressively towards prophylactic surgery.

The strengths of our study are that we addressed short-comings of previous reports in a large representative data set of the United States population. We handled the challenge of inaccurately measuring therapeutic mastectomy versus true risk-reducing mastectomies by using a person-time approach with censoring upon occurrence of breast or ovarian cancer. We utilized an interrupted time series regression analysis to test for the magnitude and statistical significance of the Jolie effect over time. We confirmed previous observations of the uptake of genetic testing and refute reports that the mastectomy or *BRCA* testing trends subsided quickly. Our analysis of monthly rates of mastectomy over a study period in excess of 20 years and with 3.33 years of follow up after the Jolie op-ed date allowed for sufficient time post-genetic testing to measure mastectomy procedures that may occur in excess of 9 months after genetic testing [29]. This is an important consideration in the interpretation of the Desai and Jenna [9] analyses as the 60-, 90-, and 180- day mastectomy rates post-genetic testing are not only limited in follow up but also subject to error and bias because health insurance claims data are subject to delays, a median of 43 days delay in a recent validation study [30] of bone metastasis diagnostic codes using electronic medical record data of breast cancer patients as the gold standard. A key limitation is that the results of *BRCA* mutation testing (and family history) were not available in the MarketScan® data, and, therefore, it remains unclear whether the rates of mastectomy among women who underwent *BRCA* testing, which increased after the Jolie op-ed publication, can be specifically extrapolated to the high-risk women found to have a mutation in either *BRCA1* or *BRCA2*.

Overall, the Angelina Jolie Effect represents a long-lasting impact of a celebrity on public health awareness with significant increases in genetic testing and mastectomy rates, which were measurable and sustained over subsequent several years.

## Electronic supplementary material

Below is the link to the electronic supplementary material.

Supplementary material 1 (DOCX 28 KB)

Supplementary material 2 (DOCX 26 KB)

Online Resource 3. STROBE Diagram [<link rid="bib12">12</link>] for Study Population Identified in MarketScan Database (1997–2016) (online only) (EPS 809 KB)
