# Supplementary material for: Risk-reducing mastectomy rates in the US: a closer examination of the Angelina Jolie effect
Source: Breast Cancer Res Treat. 2018 May 28;171(2):435–42. doi: 10.1007/s10549-018-4824-9 (PMC6096880; doi:10.1007/s10549-018-4824-9)
Supplement: Supplementary file 1 — Supplementary material 1 (DOCX 28 KB) [file 10549_2018_4824_MOESM1_ESM.docx]

**Online Resource 1. ICD-9/ICD-10 and CPT Codes for Mastectomy Procedures With or Without Reconstruction**

| **Code Type** | **Code** | **Code Description** |
| --- | --- | --- |
| ICD-9-CM Procedure | 85.41 | Unilateral simple mastectomy |
|  | 85.42 | Bilateral simple mastectomy |
|  | 85.43 | Unilateral extended simple mastectomy |
|  | 85.44 | Bilateral extended  simple mastectomy |
|  | 85.45 | Unilateral radical mastectomy |
|  | 85.46 | Bilateral radical mastectomy |
|  | 85.47 | Unilateral extended radical mastectomy |
|  | 85.48 | Bilateral extended radical mastectomy |
| CPT | 19303 | Mastectomy, simple, complete |
|  | 19180 | Mastectomy, simple, complete |
|  | 19304 | Mastectomy, subcutaneous |
|  | 19182 | Mastectomy, subcutaneous |
|  | 19200 | Mastectomy, radical, including pectoral muscles, axillary lymph nodes |
|  | 19305 | Mastectomy, radical, including pectoral muscles, axillary lymph nodes |
|  | 19220 | Mastectomy, radical, including pectoral muscles, axillary and internal mammary lymph nodes (Urban type operation) |
|  | 19306 | Mastectomy, radical, including pectoral muscles, axillary and internal mammary lymph nodes (Urban type operation) |
|  | 19307 | Mastectomy, radical, including axillary lymph nodes, with or without pectoralis minor muscles, but excluding pectoralis major muscle |
|  | 19240 | Mastectomy, radical, including axillary lymph nodes, with or without pectoralis minor muscles, but excluding pectoralis major muscle |
| ICD-9-CM Procedure | 85.33 | Unilateral subcutaneous mammectomy with synchronous implant |
|  | 85.34 | Other unilateral subcutaneous mammectomy |
|  | 85.35 | Bilateral subcutaneous mammectomy with synchronous implant |
|  | 85.36 | Other bilateral subcutaneous mammectomy |
| ICD-10-CM Procedure | 0H0T0JZ | Unilateral subcutaneous mammectomy with synchronous implant |
|  | 0H0T3JZ | Unilateral subcutaneous mammectomy with synchronous implant |
|  | 0H0U0JZ | Unilateral subcutaneous mammectomy with synchronous implant |
|  | 0H0U3JZ | Unilateral subcutaneous mammectomy with synchronous implant |
|  | 0HRT0JZ | Unilateral subcutaneous mammectomy with synchronous implant |
|  | 0HRT3JZ | Unilateral subcutaneous mammectomy with synchronous implant |
|  | 0HRU0JZ | Unilateral subcutaneous mammectomy with synchronous implant |
|  | 0HRU3JZ | Unilateral subcutaneous mammectomy with synchronous implant |
|  | 0HRV0JZ | Unilateral subcutaneous mammectomy with synchronous implant |
|  | 0HRV3JZ | Unilateral subcutaneous mammectomy with synchronous implant |
|  | 0HBT0ZZ | Other unilateral subcutaneous mammectomy |
|  | 0HBT3ZZ | Other unilateral subcutaneous mammectomy |
|  | 0HBU0ZZ | Other unilateral subcutaneous mammectomy |
|  | 0HBU3ZZ | Other unilateral subcutaneous mammectomy |
|  | 0H0V0JZ | Bilateral subcutaneous mammectomy with synchronous implant |
|  | 0H0V3JZ | Bilateral subcutaneous mammectomy with synchronous implant |
|  | 0HBV0ZZ | Other bilateral subcutaneous mammectomy |
|  | 0HBV3ZZ | Other bilateral subcutaneous mammectomy |
|  | 0HTT0ZZ | Unilateral simple mastectomy |
|  | 0HTU0ZZ | Unilateral simple mastectomy |
|  | 0HTV0ZZ | Bilateral simple mastectomy |
|  | 0HTT0ZZ | Unilateral extended simple mastectomy |
|  | 07T50ZZ | Unilateral extended simple mastectomy |
| **Code Type** | **Code** | **Code Description** |
| ICD-10-CM Procedure (continued) | 0HTU0ZZ | Unilateral extended simple mastectomy |
|  | 07T60ZZ | Unilateral extended simple mastectomy |
|  | 0HTV0ZZ | Bilateral extended simple mastectomy |
|  | 07T50ZZ | Bilateral extended simple mastectomy |
|  | 07T60ZZ | Bilateral extended simple mastectomy |
|  | 0HTT0ZZ | Unilateral radical mastectomy |
|  | 07T50ZZ | Unilateral radical mastectomy |
|  | 0KTH0ZZ | Unilateral radical mastectomy |
|  | 0HTU0ZZ | Unilateral radical mastectomy |
|  | 07T60ZZ | Unilateral radical mastectomy |
|  | 0KTJ0ZZ | Unilateral radical mastectomy |
|  | 0HTV0ZZ | Bilateral radical mastectomy |
|  | 07T50ZZ | Bilateral radical mastectomy |
|  | 07T60ZZ | Bilateral radical mastectomy |
|  | 0KTH0ZZ | Bilateral radical mastectomy |
|  | 0KTJ0ZZ | Bilateral radical mastectomy |
|  | 0HTT0ZZ | Unilateral extended radical mastectomy |
|  | 07T50ZZ | Unilateral extended radical mastectomy |
|  | 07T70ZZ | Unilateral extended radical mastectomy |
|  | 07T80ZZ | Unilateral extended radical mastectomy |
|  | 0KTH0ZZ | Unilateral extended radical mastectomy |
|  | 0HTU0ZZ | Unilateral extended radical mastectomy |
|  | 07T60ZZ | Unilateral extended radical mastectomy |
|  | 07T70ZZ | Unilateral extended radical mastectomy |
|  | 07T90ZZ | Unilateral extended radical mastectomy |
|  | 0KTJ0ZZ | Unilateral extended radical mastectomy |
|  | 0HTV0ZZ | Bilateral extended radical mastectomy |
|  | 0KTJ0ZZ | Bilateral extended radical mastectomy |
|  | 07T50ZZ | Bilateral extended radical mastectomy |
|  | 07T60ZZ | Bilateral extended radical mastectomy |
|  | 07T70ZZ | Bilateral extended radical mastectomy |
|  | 07T80ZZ | Bilateral extended radical mastectomy |
|  | 07T90ZZ | Bilateral extended radical mastectomy |
|  | 0KTH0ZZ | Bilateral extended radical mastectomy |

ICD-9-CM: International Classification of Diseases, Ninth Revision, Clinical Modification

ICD-10-CM: International Classification of Diseases, Tenth Revision, Clinical Modification

CPT: Current Procedural Terminology
