# Supplementary material for: Risk-reducing mastectomy rates in the US: a closer examination of the Angelina Jolie effect
Source: Breast Cancer Res Treat. 2018 May 28;171(2):435–42. doi: 10.1007/s10549-018-4824-9 (PMC6096880; doi:10.1007/s10549-018-4824-9)
Supplement: Supplementary file 2 — Supplementary material 2 (DOCX 26 KB) [file 10549_2018_4824_MOESM2_ESM.docx]

**Online Resource 2. ICD-9/ICD-10 Codes for Breast and Ovarian-Related Cancers**

| **Breast Cancer** |  |  |
| --- | --- | --- |
| **Code Type** | **Code** | **Code Description** |
| ICD-9-CM Diagnosis | 174.0 | Malignant neoplasm of nipple and areola of female breast |
|  | 174.1 | Malignant neoplasm of central portion of female breast |
|  | 174.2 | Malignant neoplasm of upper-inner quadrant of female breast |
|  | 174.3 | Malignant neoplasm of lower-inner quadrant of female breast |
|  | 174.4 | Malignant neoplasm of upper-outer quadrant of female breast |
|  | 174.5 | Malignant neoplasm of lower-outer quadrant of female breast |
|  | 174.6 | Malignant neoplasm of axillary tail of female breast |
|  | 174.8 | Malignant neoplasm of other specified sites of female breast |
|  | 174.9 | Malignant neoplasm of breast (female), unspecified |
| ICD-10-CM Diagnosis | C50.011 | Malignant neoplasm of nipple and areola, right female breast |
|  | C50.012 | Malignant neoplasm of nipple and areola, left female breast |
|  | C50.019 | Malignant neoplasm of nipple and areola, unspecified female breast |
|  | C50.111 | Malignant neoplasm of central portion of right female breast |
|  | C50.112 | Malignant neoplasm of central portion of left female breast |
|  | C50.211 | Malignant neoplasm of upper-inner quadrant of right female breast |
|  | C50.212 | Malignant neoplasm of upper-inner quadrant of left female breast |
|  | C50.219 | Malignant neoplasm of upper-inner quadrant of unspecified female breast |
|  | C50.311 | Malignant neoplasm of lower-inner quadrant of right female breast |
|  | C50.312 | Malignant neoplasm of lower-inner quadrant of left female breast |
|  | C50.319 | Malignant neoplasm of lower-inner quadrant of unspecified female breast |
|  | C50.411 | Malignant neoplasm of upper-outer quadrant of right female breast |
|  | C50.412 | Malignant neoplasm of upper-outer quadrant of left female breast |
|  | C50.419 | Malignant neoplasm of upper-outer quadrant of unspecified female breast |
|  | C50.511 | Malignant neoplasm of lower-outer quadrant of right female breast |
|  | C50.512 | Malignant neoplasm of lower-outer quadrant of left female breast |
|  | C50.519 | Malignant neoplasm of lower-outer quadrant of unspecified female breast |
|  | C50.611 | Malignant neoplasm of axillary tail of right female breast |
|  | C50.612 | Malignant neoplasm of axillary tail of left female breast |
|  | C50.619 | Malignant neoplasm of axillary tail of unspecified female breast |
|  | C50.811 | Malignant neoplasm of overlapping sites of right female breast |
|  | C50.812 | Malignant neoplasm of overlapping sites of left female breast |
|  | C50.819 | Malignant neoplasm of overlapping sites of unspecified female breast |
|  | C50.911 | Malignant neoplasm of unspecified site of right female breast |
|  | C50.912 | Malignant neoplasm of unspecified site of left female breast |
|  | C50.919 | Malignant neoplasm of unspecified site of unspecified female breast |

| **Ovarian Cancer** |  |  |
| --- | --- | --- |
| **Code Type** | **Code** | **Code Description** |
| ICD-9-CM Diagnosis | 183.0 | Malignant Neoplasm of ovary |
|  | 183.2 | Malignant neoplasm of fallopian tube |
|  | 158.8 | Malignant neoplasm of specified parts of peritoneum |
| ICD-10-CM Diagnosis | C56.1 | Malignant neoplasm of right ovary |
|  | C56.2 | Malignant neoplasm of left ovary |
|  | C56.9 | Malignant neoplasm of unspecified ovary |
|  | C57.00 | Malignant neoplasm of unspecified fallopian tube |
|  | C57.0 | Malignant neoplasm of fallopian tube |
|  | C57.01 | Malignant neoplasm of right fallopian tube |
|  | C57.02 | Malignant neoplasm of left fallopian tube |
|  | C48.1 | Malignant neoplasm of specified parts of peritoneum |
|  | C48.2 | Malignant neoplasm of peritoneum, unspecified |

ICD-9-CM: International Classification of Diseases, Ninth Revision, Clinical Modification

ICD-10-CM: International Classification of Diseases, Tenth Revision, Clinical Modification

CPT: Current Procedural Terminology
